# EUS-Guided Gallbladder Drainage of Inoperable Malignant Distal Biliary Obstruction by Lumen-Apposing Metal Stent: Systematic Review and Meta-Analysis

**DOI:** 10.3390/cancers17121983

**Published:** 2025-06-13

**Authors:** Tawfik Khoury, Moaad Farraj, Wisam Sbeit, Pietro Fusaroli, Giovanni Barbara, Cecilia Binda, Carlo Fabbri, Maamoun Basheer, Sarah Leblanc, Fabien Fumex, Rodica Gincul, Anthony Yuen Bun Teoh, Jérémie Jacques, Bertrand Napoléon, Andrea Lisotti

**Affiliations:** 1 Department of Gastroenterology, Galilee Medical Center, Nahariya 22100, Israel; wisams@gmc.gov.il (W.S.); maamounb@gmc.gov.il (M.B.); 2 AZrieli Faculty of Medicine, Bar-Ilan University, Safed 1311502, Israel; 3 Department of Gastroenterology, Hôpital Privé Jean Mermoz, Ramsay Santé, 69008 Lyon, France; sarahleblanc34@hotmail.com (S.L.); fabien.fumex@wanadoo.fr (F.F.); rodica_h13@yahoo.fr (R.G.); dr.napoleon@wanadoo.fr (B.N.); lisotti.andrea@gmail.com (A.L.); 4 Department of General Surgery, Galilee Medical Center, Nahariya, Azrieli Faculty of Medicine, Bar-Ilan University, Safed 1311502, Israel; farrajm@gmc.gov.il; 5 Gastroenterology Unit, Hospital of Imola, University of Bologna, 40026 Bologna, Italy; pietro.fusaroli@unibo.it; 6 Department of Medical and Surgical Sciences (DIMEC), University of Bologna, 40126 Bologna, Italy; giovanni.barbara@unibo.it; 7 Division of Internal Medicine, IRCCS Azienda Ospedaliero-Universitaria di Bologna, 40138 Bologna, Italy; 8 Gastroenterology and Digestive Endoscopy Unit, Forlì-Cesena Hospitals, AUSL Romagna, 47100 Forlì-Cesena, Italy; cecilia.binda@auslromagna.it (C.B.); carlo.fabbri@auslromagna.it (C.F.); 9 Department of Surgery, Prince of Wales Hospital, The Chinese University of Hong Kong, Hong Kong SAR, China; anthonyteoh@surgery.cuhk.edu.hk; 10 Service d’Hépato-Gastroentérologie, CHU Dupuytren Limoges, 87000 Limoges, France; jeremiejacques@gmail.com

**Keywords:** gallbladder drainage, biliary obstruction, efficacy, safety, drainage

## Abstract

**Objective**: Endoscopic ultrasound-guided gallbladder drainage (EUS-GBD) has emerged as a promising alternative for biliary decompression in patients with malignant distal biliary obstruction (MDBO), used either as a first-line approach or after other interventions have failed. This study aimed to evaluate the aggregated efficacy and safety of EUS-GBD in this patient population. **Methods**: A comprehensive literature search was carried out across PubMed/Medline, Embase, and Cochrane databases up to 9 January 2024, to identify studies reporting outcomes of EUS-GBD in MDBO cases. The primary endpoint assessed was clinical success, while secondary endpoints included technical success and the incidence of adverse events (AEs). Pooled outcomes were calculated using a random-effects model and presented with 95% confidence intervals (CIs). **Results**: Seven studies encompassing a total of 193 patients were included in the analysis. The combined clinical success rate for EUS-GBD was 88.1% [95% CI: 78.9–94.9%], while the technical success rate was 99.2% [95% CI: 97.5–100%]. The overall AE rate was 13.7% [95% CI: 9.3–18.8%], with the majority being mild to moderate in severity; no fatal complications were reported. Subgroup analyses indicated that use of smaller lumen-apposing metal stents (LAMS) (<15 mm) was associated with slightly higher clinical success (93.3% [95% CI: 72.4–99.9%]) compared to larger stents (≥15 mm) (87.1% [95% CI: 78.8–93.5%]), and a marginally lower rate of AEs (12.3% [95% CI: 6.4–19.7%] vs. 15.2% [95% CI: 6.5–26.6%]). **Conclusions**: EUS-GBD demonstrates excellent technical performance, high clinical efficacy, and a manageable safety profile in patients with MDBO and a patent cystic duct.

## Simple Summary

Malignant distal biliary obstruction (MDBO) is a challenging condition often requiring biliary drainage for symptom relief. Endoscopic ultrasound-guided gallbladder drainage (EUS-GBD) has recently emerged as an alternative technique, especially when conventional approaches fail or are not feasible. This study systematically reviewed and analyzed the available evidence on the efficacy and safety of EUS-GBD in this context. Results from seven studies involving 193 patients showed high technical and clinical success rates, with an acceptable rate of mostly mild to moderate adverse events. These findings support the role of EUS-GBD as a viable drainage option in selected MDBO patients with a patent cystic duct.

## 1. Introduction

Biliary drainage in patients with malignant distal biliary obstruction (MDBO) is most commonly managed through endoscopic retrograde cholangiopancreatography (ERCP) with stent placement, which remains the standard therapeutic approach [1]. In case of ERCP failure, several rescue strategies could be adopted to achieve adequate biliary drainage in patients with MDBO, including either the EUS-guided or percutaneous rendezvous technique, EUS-guided choledocho-duodenostomy (EUS-CDS), EUS-guided antegrade stenting, and EUS-guided hepatico-gastrostomy (EUS-HGS). EUS-guided rendezvous has an 81% overall success rate; however, it is associated with a 10% adverse events (AEs) rate [2]. Similarly, EUS-guided choledochoduodenostomy and hepaticogastrostomy have shown technical success rates of >90%, with AE rates ranging from 8% to 14% in recent meta-analyses [3,4], although some earlier studies reported higher figures. EUS-guided gallbladder drainage (EUS-GBD) has been shown to be an effective and safe therapeutic treatment in high-risk surgical patients with acute cholecystitis. A recent meta-analysis including 27 articles (1004 patients) showed pooled technical success of 98% (95% CI 96.3, 99.3), with overall clinical success of 95.4% (95% CI 92.8, 97.5), and procedure-related adverse events of 14.8% (95% CI 8.8, 21.8) [5]. Recently, several studies reported the efficacy and safety of EUS-GBD for biliary drainage in patients with MDBO, either as a rescue strategy or first-line approach. The aim of our study was to assess the pooled performance of EUS-GBD for MDBO. The primary objective was to evaluate the clinical success rate, while technical success rate, and incidence of AEs were secondary objectives. Finally, a subgroup analysis for the primary outcome and for AEs were conducted.

## 2. Materials and Methods

This systematic review and meta-analysis were conducted and documented in alignment with the PRISMA guidelines [6].

### 2.1. Eligibility Criteria

We included peer-reviewed articles published in English, with no restriction on publication date, up to the final search on 9 January 2024 that fulfilled the following criteria: (a) population: adult individuals diagnosed with malignant distal biliary obstruction (MDBO); (b) intervention: endoscopic ultrasound-guided gallbladder drainage (EUS-GBD), applied either as a primary treatment or a rescue strategy; (c) outcomes: the primary endpoint was clinical success, while secondary endpoints included technical success and both the overall and severe adverse event (AE) rates. Exclusion criteria were: (a) case reports; (b) studies omitting data on the primary outcome; (c) abstracts not published in a peer-reviewed journal within two years.

### 2.2. Search Methods

A comprehensive search of PubMed/Medline, Embase, and Cochrane databases was performed through 9 January 2024. The search was limited to English-language publications and was independently carried out by two reviewers (TK and AL). The search terms included: (“EUS-guided” OR “EUS” OR “Endosonography” [Mesh] OR “Endoscopic ultrasound”) AND (cholecysto-duodenostomy OR cholecysto-gastrostomy OR lumen-apposing metal stent OR Hot Axios OR Spaxus OR Hot-Spaxus) AND (malignant distal biliary obstruction OR MDBO). References from the studies included were also screened for potential inclusion. All study designs were considered except case reports and abstracts without full-text publication. For multiple reports from the same cohort, the most recent publication was selected.

### 2.3. Quality Appraisal

Each study was independently assessed by two reviewers (T.K. and W.S.) using the Newcastle–Ottawa Scale (NOS). Any discrepancies regarding the data extraction or quality assessment were rare (inter-reviewer disagreement < 10%) and were resolved through consensus or, when needed, adjudicated by a third investigator (A.L.). NOS cut-off was based on total score: low quality: <6 points; moderate quality: 6–7 points; high quality: ≥8 points.

### 2.4. Data Collection

The extracted information included the name of the first author, year of publication, study sample size, underlying cause of MDBO, stent type, success rates (clinical and technical), incidence and severity of AEs, and procedure-related mortality.

For studies in which adverse events (AEs) were reported without specification of type or timing, events were included in the pooled analysis only if explicitly stated by the authors and were categorized as ‘not specified’ in the relevant tables.

### 2.5. Definition of Outcomes

The primary outcome, clinical success, was defined as either a ≥50% reduction in serum total bilirubin or a decrease to below 3 mg/dL within two weeks [7]. This was chosen as the primary endpoint due to its clinical significance. Secondary outcomes included (1) technical success, characterized by accurate deployment of the lumen-apposing metal stent (LAMS) into the gallbladder; and (2) AE rate, categorized by severity per the American Society of Gastrointestinal Endoscopy (ASGE) lexicon [8]. Severe AEs included those resulting in extended hospitalization (>10 days), ICU admission (>1 night), surgical intervention, or lasting disability.

### 2.6. Statistical Analysis

Pooled estimates of EUS-GBD performance were calculated as proportions with 95% confidence intervals (CIs) for dichotomous outcomes. The meta-analysis employed a DerSimonian and Laird random-effects model. Heterogeneity was quantified using the I^2^ statistic, with thresholds set at <30% (low), 30–60% (moderate), and >60% (high). Funnel plot visualization and Egger’s test were used to assess potential publication bias. Subgroup analyses were conducted based on (1) sample size (<35 participants), (2) study design (retrospective vs. prospective), (3) publication year (pre-2023 vs. 2023 and later), (4) LAMS type (Hot Axios vs. Hot Spaxus), (5) LAMS size (≥15 mm vs. <15 mm), and (6) indication (post-ERCP failure vs. primary drainage). Statistical analyses were performed using MedCalc^®^ version 20.115 (MedCalc Software Ltd., Ostend, Belgium; https://www.medcalc.org; accessed on 1 January 2020), with statistical significance set at a two-sided *p*-value < 0.05.

## 3. Results

**Figure 1:**
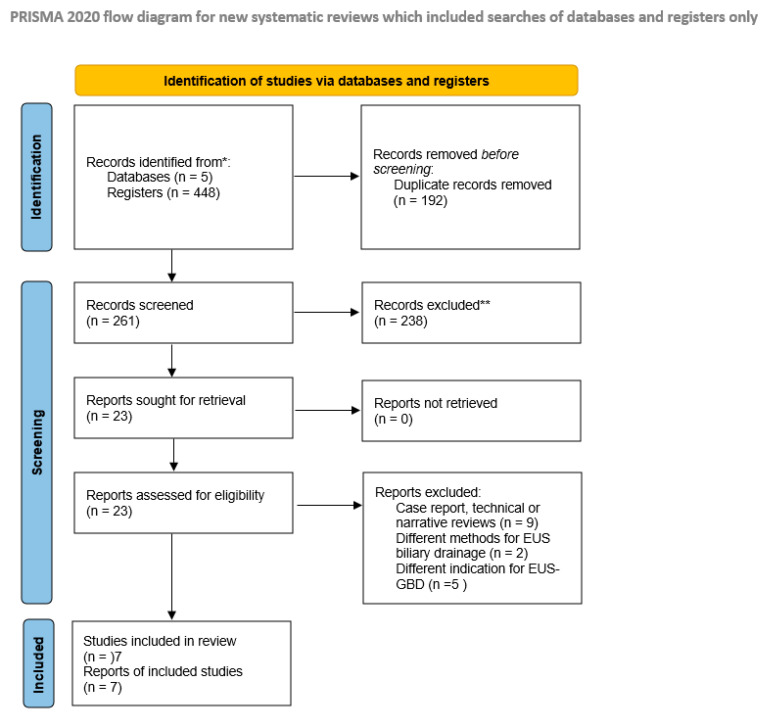
PRISMA flow diagram of the study. * Records identified through primary electronically search ** Studies excluded, as they were obviously irrelevant studies (n = 237), and abstract without full-text manuscript that was published within 3 years (n = 1).

***Literature search and study characteristics*.** Figure 1 demonstrates the literature search according to the PRISMA reporting form. Overall, the literature search yielded 453 potentially relevant studies. Twenty-three publications were fully reviewed after preliminary screening of titles and abstracts. Finally, six papers and one congress abstract were included in this meta-analysis [9,10,11,12,13,14,15]. Table 1 summarizes the studies’ characteristics.

Table 2 summarizes the methodological quality evaluation of the studies included. According to Newcastle–Ottawa Scale (NOS) for non-randomized studies. Included studies were classified as overall low (no. 2) or medium (no. 5) quality due to the lack of a comparator group in all studies and moderate concerns in the selection criteria (5 studies) and outcomes identification (2 studies). Moreover, the risk of bias was assessed. Most studies had moderate selection bias (5 studies), which was similar for performance bias (4 studies), while detection bias was low in 3 studies and moderate in 3 studies. For attribution bias, 6 studies had moderate bias. Table 3 demonstrates risk of biases across studies.

**Figure 2:**
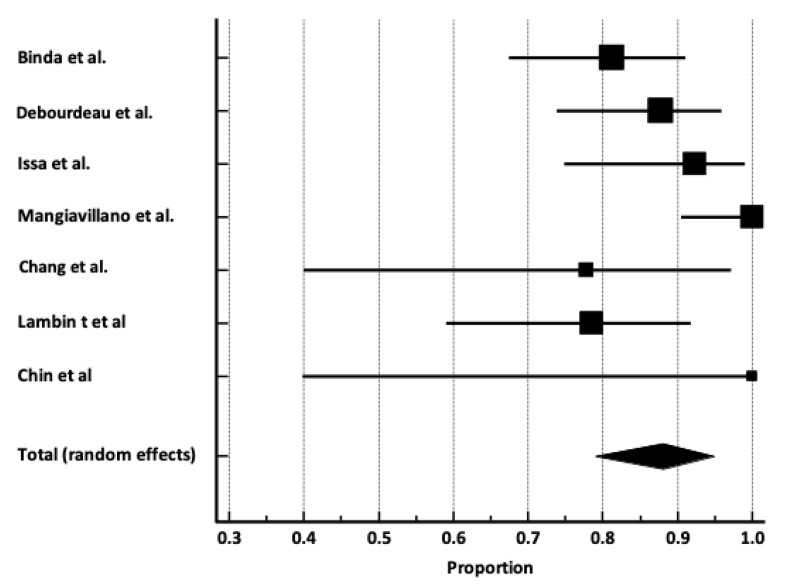
Forrest plot reporting pooled estimates for clinical success rate [9,10,11,12,13,14,15].

***Pooled clinical success rate***. The pooled clinical success rate was 88.1% [95% C.I. 78.9–94.9%], shown in Figure 2, with moderate heterogenicity (I^2^ 65.2%) (Table 4). No publication bias was observed (Supplementary Figure S1a) (Egger’s test *p* = 0.95).

**Figure 3:**
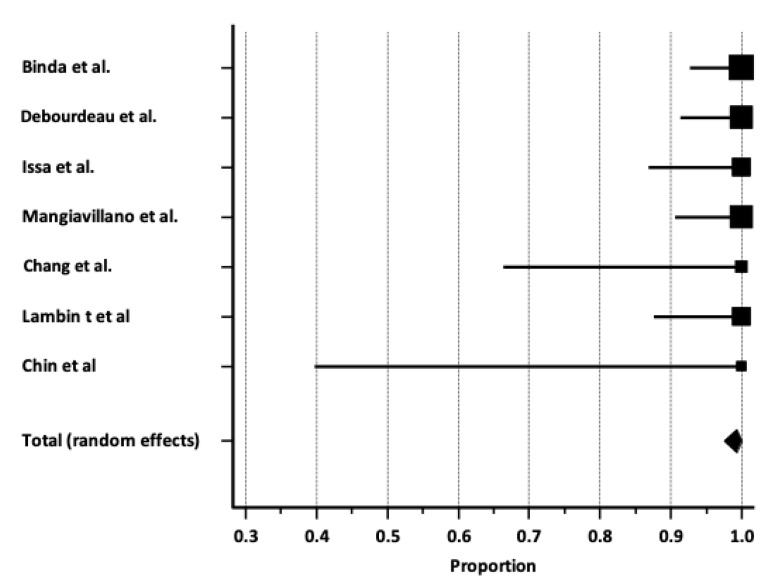
Forrest plot reporting pooled estimates for technical success rate [9,10,11,12,13,14,15].

Pooled technical success rate. Pooled technical success with EUS-GB was 99.2%; [95% C.I. 97.5–100%], as shown in Figure 3, with low heterogeneity (I^2^ 0%) (Table 4). A potential publication bias (Supplementary Figure S1b) was observed and confirmed by Egger’s test (*p*< 0.001).

**Figure 4:**
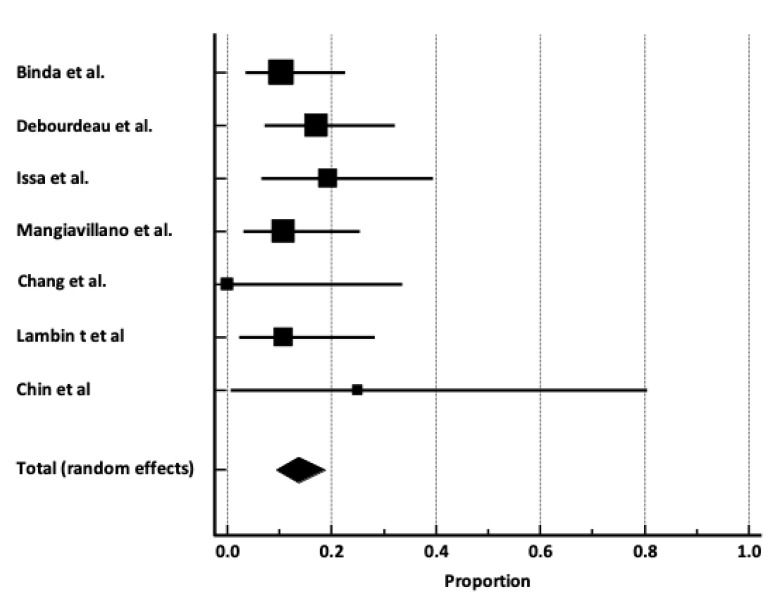
Forrest plot reporting pooled incidence of adverse events rate [9,10,11,12,13,14,15].

Safety profile. The overall pooled incidence of AEs was 13.7% [95% C.I. 9.3–18.8%], with no heterogenicity (I^2^ 0%) (Figure 4); no publication bias (Supplementary Figure S1c) was observed (Egger’s test P = 0.95). The pooled rate of early and late AEs were 4.5% [95% C.I. 2–7.9%] and 9.5% (95% C.I. 5.7–14.3%] (Supplementary Figure S2a,b), with a low level of heterogenicity (I^2^ 5.1%, and I^2^ 9.2%), respectively. No publication bias was observed (Table 3). Most of the AEs were mild to moderate, as the pooled rates of mild, moderate, and severe AEs were 4.2% [95% C.I. 0.4–11.8%], 7.6% [95% C.I. 4.3–11.6%], and 1.1% [95% C.I. 0.1–3.1%], with no heterogenicity for moderate and severe AEs (I^2^ 0%) (Supplementary Figure S3a–c), and high-level of heterogeneity for mild AEs (I^2^ 73.8%). No publication bias was observed.

Studies including up to 35 patients (4 studies, 67 patients) revealed a pooled clinical success rate of 84.3% [95% C.I. 74.8–91.8%], with a low level of heterogenicity (I^2^ 1.9%), while studies including ≥ 35 patients (3 studies, 126 patients), showed pooled clinical success of 91% [95% C.I. 75.1–99.2%], with high heterogenicity (I^2^ 84.2%) (Table 5). EUS-GBD performed with the 10 mm LAMS had a pooled clinical success of 93.3% [95% C.I. 72.4–99.9%], and 87.1% [95% C.I. 78.8–93.5%] with a ≥15 mm LAMS diameter. Additionally, the pooled success was 84.4% [95% C.I. 78.3–89.7%] in EUS-GBD performed after failed ERCP (5 studies, 147 patients), and 92.3% [95% C.I. 57.5–97.8%] performed as the primary intervention (2 studies, 46 patients). We were not able to assess the subgroup analysis on the LAMS type (Axios vs. Spaxus) as there was only one study that reported the use of Spaxus stent.

Subgroup analysis for the incidence of adverse events. The incidence of AEs rate was similar between studies including <35 patients (4 studies, 67 patients), with a pooled AEs rate of 14% [95% C.I. 6.2–24.3%], and studies including ≥35 patients (3 studies, 126 patients), with pooled rate of 13.4% [95% C.I. 8.1–19.8%]. The pooled AEs rate with hot Axios was 14.1% [95% C.I. 9.2–19.9%] (6 studies, 156 patients), compared to 10.8% [95% C.I. 3–18.8%] with hot Spaxus (1 study, 37 patients). Additionally, the AEs rate was higher among patients who underwent EUS-GBD after failed ERCP in 15.2% [95% C.I. 9.9–21.3%] vs. 9.0% [95% C.I. 2.1–20.1%] in EUS-primary GBD (Table 6).

## 4. Discussion

This systematic review included five retrospective, one prospective, and one conference abstract reporting the performance of EUS-GBD in 193 patients suffering from MDBO. The quantitative results of our analysis show a high pooled clinical success rate (88.1%), with an optimal technical success rate (99.2%). While the pooled incidence of AEs was 13.7%, most of them were mild to moderate according to the American Society for Gastrointestinal Endoscopy lexicon, with no periprocedural mortality, and only one case of intra-procedural LAMS dislodgment. Table 7 demonstrates the AEs of the studies included. On subgroup analysis for the AEs, EUS-GBD performed after failed ERCP was associated with a higher AEs rate of (15.2% vs. 9%) compared to EUS-GBD performed as a primary drainage modality. The higher AEs after a failed ERCP mainly derived from the increased rate of post-ERCP pancreatitis.

The substantial heterogeneity observed in the ≥35 patient subgroup (I^2^ = 84.2%) likely reflects real-world variability across larger studies in terms of operator experience, patient selection, and procedural technique. Differences in cystic duct patency assessment, tumor location, and choice of LAMS size or design may have contributed to the observed dispersion of clinical success rates. Moreover, multicenter or more recent studies may capture a broader range of practice patterns, further amplifying heterogeneity despite overall favorable outcomes.

The reported optimal technical success rate (up to 100%) may be overestimated mainly due to the retrospective design of most studies (EUS-GBD was started after the identification of an enlarged gallbladder close to the stomach or duodenum). Notably, there was a potential publication bias for technical success rate (Egger’s test < 0.001), this finding could be explained by unpublishing data showing the unsuccessful cases of EUS-GBD, which might affect the outcomes assessed, thus leading to this bias. Moreover, the use of large (≥15 mm) LAMS led to a slight increase in incidence of AE and reduced clinical success.

Our results provide considerable innovative information, thus extending the role of EUS-GBD in MDBO and not only limiting it to acute cholecystitis, with almost perfect technical success and very good clinical success.

A previous meta-analysis demonstrated a clinical success rate of EUS-GBD of 85% [16]. Our results showed an even higher clinical success rate of 88.1%.

A free cystic duct is a prerequisite for successful EUS-GBD [17] and should be assessed by cross-sectional abdominal imaging before EUS-GBD. All studies in our meta-analysis included patients with a patent cystic duct. However, Mangiavillano et al. reported about two patients with cystic duct involvement [15]. Another study including patients suffering from MDBO suggested that the presence of tumors close (within 1 cm) to the cystic duct implant should be considered a contraindication for EUS-GBD [18]. Therefore, EUS-GBD should be considered a viable approach for biliary drainage when the cystic duct can be followed on EUS to assess patency and there is at least 1 cm from the proximal portion of the tumor and hepato-cystic junction.

Despite the small number of studies included in this systematic review, we performed two further analyses (Table 5 and Table 6). The sensitivity analysis conducted on the main outcome suggests that studies conducted more recently (after 2023) and larger studies (≥35 patients) account for the higher clinical success rate. This is probably due to the increased experience with EUS-GBD and better patient selection. Subgroup analyses assessing the incidence of AEs in different groups show that the use of large (≥15 mm) LAMS led to a slight increase in incidence of AEs. A previous study stated that the preferred size for EUS-GBD for biliary drainage is the smaller caliber LAMS (10 mm, or 8 mm if available), while larger LAMS (≥15 mm) may be preferred if peroral cholecystoscopic interventions are planned [19]. We hypothesized that the use of larger LAMS could lead to increased wall trauma and stent-related complications, especially food impaction. Conversely, smaller LAMS may better match the gallbladder anatomy, minimizing bile leakage and tissue disruption.

Unfortunately, we were not able to compare different LAMS designs (i.e., Hot-Spaxus vs. Hot-Axios) since just one study by Mangiavillano et al. reported about the former [15]. The main differences between the two LAMS designs are based on size (caliber and length available), the presence of cover portions, the capability of apposing the two lumens, and the less traumatic peripheral portions. However, a recent propensity-matched study by Mangiavillano et al. showed that in patients with pancreatic fluid collections, bleeding requiring transfusion and/or intervention occurred significantly more frequently with use of the Hot-Axios stent than with the Spaxus stent [20]. Therefore, further studies are needed to assess possible differences in AE incidence among patients suffering from MDBO undergoing EUS-GBD, according to the LAMS design.

No included study reported about the prevalence of gallbladder stones; therefore, this study does not provide robust evidence on this field, even if some authors considered the presence of stones a possible contraindication for EUS-GBD in this setting [10].

Several studies and the most recent guidelines [21,22,23,24,25,26] suggest that EUS-GBD should be preferentially performed through a trans-duodenal approach in high-risk surgical patients with acute cholecystitis due to a reduced risk of long-term adverse events and stent dysfunction. To date, no robust data is available comparing the trans-gastric and trans-duodenal approach for EUS-GBD in patients with DMBO.

Finally, although the included studies were not comparative, EUS-GBD clinical and technical success were not inferior to EUS-guided biliary drainage and EUS-guided hepaticogastrostomy, with a slightly lower AEs rate among patients with MDBO [3,27,28,29,30]. Therefore, EUS-GBD could be added to the therapeutic armamentarium as an effective and safe intervention in this population of patients.

Our meta-analysis has several limitations. Six of the seven included studies were retrospective in nature and could have contributed to an overestimation of the technical success rate. In particular, the decision to proceed with EUS-GBD was frequently based on favorable anatomical conditions, such as a distended gallbladder in close proximity to the gastrointestinal lumen, which may not be consistently reported in failed or technically challenging cases. As such, the high pooled technical success rate observed (99.2%) may be overestimated, reflecting a preselection of optimal candidates rather than the broader clinical population. Two studies graded as low quality were still included as they provided unique data not reported in other cohorts, and because all studies, regardless of quality, were evaluated in subgroup and sensitivity analyses to assess their influence on pooled estimates. Moreover, most studies have been conducted in tertiary-referral centers by highly experienced operators. The optimal performance of EUS-GBD in other settings (i.e., acute cholecystitis) cannot be automatically translated to this setting when operators from low-volume centers were involved. Moreover, no study specified if included patients presented with concomitant acute cholecystitis and no study specified the relative outcomes of patients drained via the trans-gastric or trans-duodenal approaches; the lack of these data impairs the capability of performing further analysis to address those relevant questions. Finally, to date, most studies have been conducted with the use of one LAMS design (namely Hot-Axios stent, Boston Scientific); the results of EUS-GBD performed with other LAMS designs should be accurately studied before drawing any conclusions.

## 5. Conclusions

This meta-analysis supports the potential of EUS-guided gallbladder drainage (EUS-GBD) as a highly effective option for biliary drainage in patients with malignant distal biliary obstruction (MDBO). However, unlike other EUS-guided biliary drainage techniques, such as choledochoduodenostomy (EUS-CDS) and hepaticogastrostomy (EUS-HGS), EUS-GBD should only be considered when endoscopic ultrasound confirms a patent cystic duct and the tumor does not involve its insertion. Notably, our findings raise new considerations, including the possible advantage of smaller (10 mm) lumen-apposing metal stents (LAMS), although these observations require validation through larger, prospective studies. Further head-to-head comparisons between EUS-GBD and conventional EUS-BD approaches are necessary to establish the most appropriate strategy in cases where ERCP fails. Despite promising outcomes, EUS-GBD should not yet be adopted as a standard treatment until randomized controlled trials provide more definitive evidence.

## Figures and Tables

**Table 1:** Baseline study characteristics.

Variables	Binda et al. [9]	Debourdeau et al. [12]	Issa et al. [13]	Mangiavillano et al. [15]	Chang et al. [10]	Lambin et al. [14]	Chin et al. [11]
Journal	Gastrointestinal endoscopy	Digestive endoscopy	Endoscopy	Digestive endoscopy	Endoscopy international open	Abstract	Endoscopy international open
Year published	2023	2024	2021	2024	2018	2021	2020
Design of study	Retrospective	Retrospective	Retrospective	Prospective	Retrospective	Retrospective	Retrospective
Cause of MDBO	Pancreatic cancer Cholangiocarcinoma Ampullary cancer Others	Pancreatic cancer Cholangiocarcinoma Ampullary cancer Others	NR	Pancreatic cancer Cholangiocarcinoma Ampullary cancer	Pancreatic cancer	Pancreatic cancer Cholangiocarcinoma Others	NR
Patients, n	48	41	26	37	9	28	4
Type of LAMS	Hot Axios	Hot Axios	Hot Axios	Hot Spaxus	Hot Axios	Hot Axios	Hot Axios
Route of drainage	Trans-gastric (58.3%) Trans-duodenal (41.7%)	Trans-gastric (100%)	Trans-gastric (46%) Trans-duodenal (54%)	Trans-gastric (40.6%) Trans-duodenal (59.4%)	Trans-gastric (44.4%) Trans-duodenal (55.6%)	NR	Trans-duodenal (100%)
Follow-up (median)	122	170	990	120	130.7	108	237

Abbreviations: NR: Not reported.

**Table 2:** Quality assessment of included studies according to the Newcastle–Ottawa Scale (NOS) for non-randomized studies.

Reference	Selection	Comparability	Outcome	Overall
Binda et al. [9]	Medium	Not assessable	High	Medium
Debourdeau et al. [12]	High	Not assessable	High	Medium
Issa et al. [13]	High	Not assessable	High	Medium
Mangiavillano et al. [15]	High	Not assessable	High	Medium
Chang et al. [10]	Medium	Not assessable	High	Medium
Lambin t et al. [14]	Medium	Not assessable	Medium	Low
Chin et al. [11]	Medium	Not assessable	Medium	Low

**Table 3:** Risk of bias across studies.

Reference	Selection Bias	Performance Bias	Detection Bias	Attribution Bias
Binda et al. [9]	Moderate	Moderate	Moderate	Moderate
Debourdeau et al. [12]	Moderate	High	Moderate	Moderate
Issa et al. [13]	Moderate	Moderate	Moderate	Moderate
Mangiavillano et al. [15]	Low	Low	Low	Moderate
Chang et al. [10]	High	High	High	High
Lambin t et al. [14]	Moderate	Moderate	Low	Moderate
Chin et al. [11]	Moderate	Moderate	Low	Moderate

**Table 4:** Pooled performance of endoscopic ultrasound-guided gallbladder drainage (EUS-GBD) for biliary drainage in patients suffering from malignant distal biliary obstruction (MDBO).

	No. Studies (Population)	Pooled Estimates [95% CI] Random Effect Model	Heterogeneity (I^2^)	Egger’s Test (*p* Value)
Technical success rate	7 studies (193 patients)	99.2% [97.5–100%]	0.0%	<0.001
Clinical success rate	7 studies (193 patients)	88.1% [78.9–94.9%]	65.2%	0.95
Adverse event rate	7 studies (193 patients)	13.7% [9.3–18.8%]	0.0%	0.97
**AEs timing**				
• Early AEs rate	7 studies (193 patients)	4.5% [2.0–7.9%]	5.1%	0.48
• Delayed AEs rate	7 studies (193 patients)	9.5% [5.7–14.3%]	9.2%	0.42
**AEs severity**				
• Mild AEs rate	7 studies (193 patients)	4.2% [0.4–11.8%]	73.8%	0.67
• Moderate AEs rate	7 studies (193 patients)	7.6% [4.3–11.6%]	0.0%	0.32
• Severe AEs rate	7 studies (193 patients)	1.1% [0.1–3.1%]	0.0%	0.16

Abbreviations: AEs—adverse events; CI—confidence interval.

**Table 5:** Subgroup analysis for main outcome measure (clinical success rate).

*Random Effect Model*	No. Studies (Population)	Pooled Estimates [95% Confidence Interval]	Heterogeneity (I^2^)
***Study population***			
Study population < 35 patients	4 studies (67 patients)	84.3% [74.8–91.8%]	1.9%
Study population ≥ 35 patients	3 studies (126 patients)	91.0% [75.1–99.2%]	84.2%
***Study design***			
Retrospective design	6 studies (156 patients)	83.9% [77.9–89.2%]	0.0%
Prospective design	1 study (37 patients)	100% [90.5–100%%]	N/A
***Publication type***			
Full-text article	6 studies (165 patients)	86.5% [79.1–90.3%]	3.2%
***Study period***			
Before 2023	4 studies (67 patients)	84.3% [74.8–91.8%]	1.9%
2023–2024	3 studies (126 patients)	91.0% [75.1–99.2%]	84.2%
***LAMS type***			
Hot-Axios stent	6 studies (156 patients)	83.9% [77.9–89.2%]	0.0%
Hot-Spaxus stent	1 study (37 patients)	100% [90.5–100%%]	N/A
***LAMS diameter***			
Large (≥15 mm) LAMS	3 studies (76 patients)	87.1% [78.8–93.5%]	0.0%
Small (<15 mm) LAMS	3 studies (89 patients)	93.3% [72.4–99.9%]	83.4%
***Gallbladder drainage intention***			
After ERCP failure	5 studies (147 patients)	84.4% [78.3–89.7%]	0.0%
EUS-GBD primary drainage	2 studies (46 patients)	92.3% [57.5–97.8%]	83.7%

Abbreviations: N/A not assessable; ERCP: endoscopic retrograde cholangio-pancreatography; EUS-GBD: endoscopic ultrasound-guided gallbladder drainage.

**Table 6:** Subgroup analysis for the incidence of adverse events.

	No. Studies (Population)	Incidence of AEs [95% CI] Random Effect Model
***Study population***		
Study population < 35 patients	4 studies (67 patients)	14.0% [6.2–24.3%]
Study population ≥ 35 patients	3 studies (126 patients)	13.4% [8.1–19.8%]
***LAMS type***		
Hot-Axios stent	6 studies (156 patients)	14.1% [9.2–19.9%]
Hot-Spaxus stent	1 study (37 patients)	10.8% [3.0–18.8%%]
***LAMS diameter***		
Large (≥15 mm) LAMS	3 studies (76 patients)	15.2% [6.5–26.6%]
Small (<15 mm) LAMS	3 studies (89 patients)	12.3% [6.4–19.7%]
***Gallbladder drainage intention***		
After ERCP failure	5 studies (147 patients)	15.2% [9.9–21.3%]
EUS-GBD primary drainage	2 studies (46 patients)	9.0% [2.1–20.1%]

Abbreviations: AEs: adverse events; ERCP: endoscopic retrograde cholangiopancreatography; EUS-GBD: endoscopic ultrasound-guided gallbladder drainage.

**Table 7:** Adverse events details of the included studies.

Reference	Number of AEs	Type of AEs	Severity
**Binda et al. [9]**			
Early (intraprocedural)	3	Bleeding (2), dislodgment (1)	Mild–moderate
Late (>15 days)	2	Buried stent (1), occlusion (1)	Moderate
**Debourdeau et al. [12]**			
Early (within 24 h)	4	Bleeding (2), dislodgment (1), bacteremia (1)	Mild–severe
Late (after 24 h)	3	Bleeding (1), stent obstruction (2)	Moderate
**Issa et al. [13]**			
Early (within 24 h)	0	-	-
Late (after 24 h)	5	Food impaction (3), bleeding (2)	Moderate
**Mangiavillano et al. [15]**			
Early (within 48 h)	1	Bleeding (1)	Moderate
Late (after 48 h)	3	Occlusion (1), cystic duct occlusion by tumor (2)	Moderate
**Chang et al. [10]**	NR	NR	NR
Early			
Late			
**Lambin T et al. [14]**			
Early (periprocedural)	0	-	-
Late (within 30 days)	3	Obstruction (1), cholangitis (1), septic shock (1)	Moderate
**Chin et al. [11]**			
Early (<30 days)	0	-	-
Late (>30 days)	1	Migration (1)	Moderate

## Data Availability

The datasets generated during and/or analyzed during the current study are available from the corresponding author on reasonable request.

## Supplementary Materials

The following supporting information can be downloaded at: https://www.mdpi.com/article/10.3390/cancers17121983/s1. Supplementary Figure S1a: Funnel plots assessment for publication bias of clinical success rate; Supplementary Figure S1b: Funnel plots assessment for publication bias of technical success rate; Supplementary Figure S1c: Funnel plots assessment for publication bias of incidence of adverse events; Supplementary Figure S2a,b: Pooled estimates for incidence of early and delayed adverse events; Supplementary Figure S3a,c: Pooled estimates for incidence of (a) mild, (b) moderate, and (c) severe adverse events.

## Abbreviations

EUS-GBD: endoscopic ultrasound-guided gallbladder drainage; MDBO: malignant distal biliary obstruction; AEs: adverse events; CI: confidence interval; LAMS: lumen-apposing metal stent; ERCP: endoscopic retrograde cholangiopancreatography; EUS-CDS: EUS-guided choledocho-duodenostomy; EUS-HGS: EUS-guided hepatico-gastrostomy; EUS-BD: endoscopic ultrasound-guided biliary drainage.
