# Supplementary figures and images for: EUS-Guided Gallbladder Drainage of Inoperable Malignant Distal Biliary Obstruction by Lumen-Apposing Metal Stent: Systematic Review and Meta-Analysis
Source: Cancers (Basel). 2025 Jun 13;17(12):1983. doi: 10.3390/cancers17121983 (PMC12190326; doi:10.3390/cancers17121983)

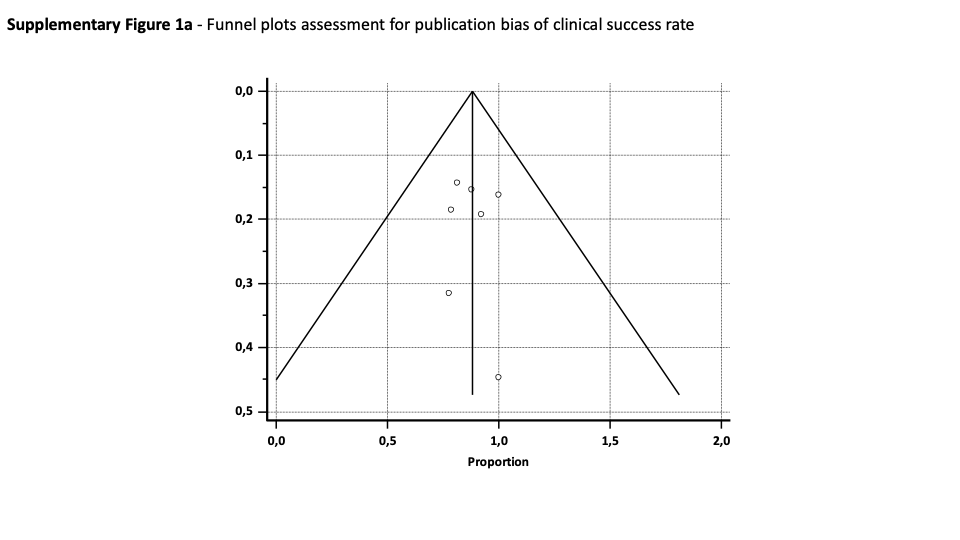

Supplement: Supplementary file 1 [file cancers-17-01983-s001.zip › Supplementary Figure S1a.tiff]

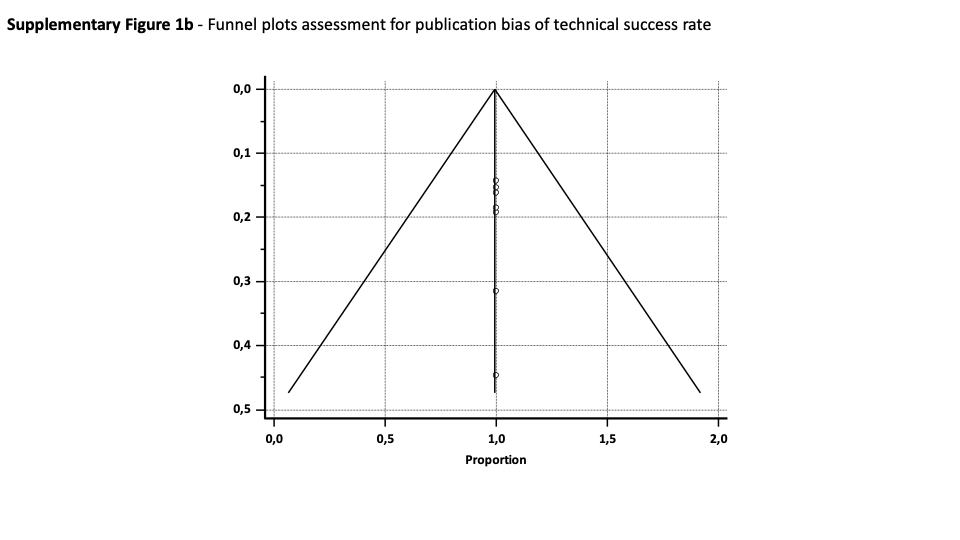

Supplement: Supplementary file 1 [file cancers-17-01983-s001.zip › Supplementary Figure S1b.tiff]

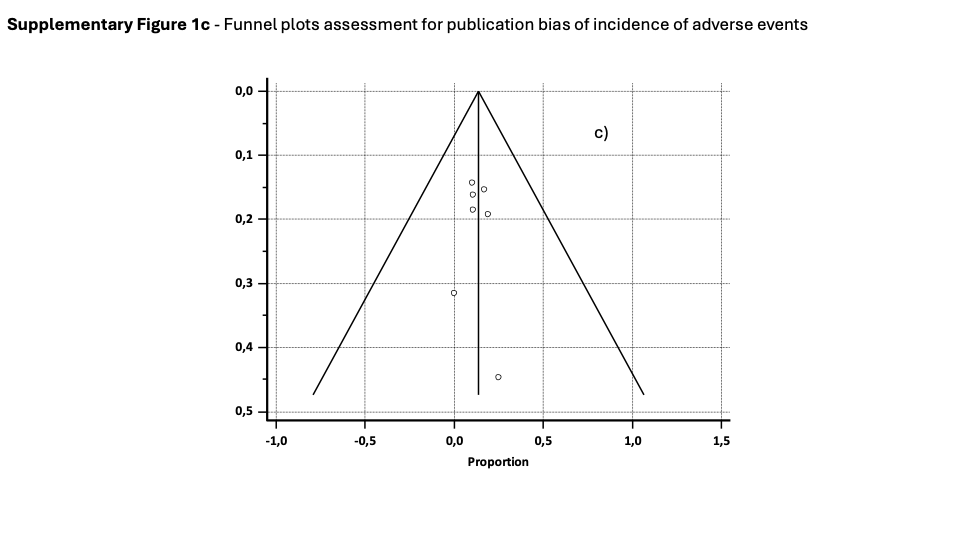

Supplement: Supplementary file 1 [file cancers-17-01983-s001.zip › Supplementary Figure S1c.tiff]

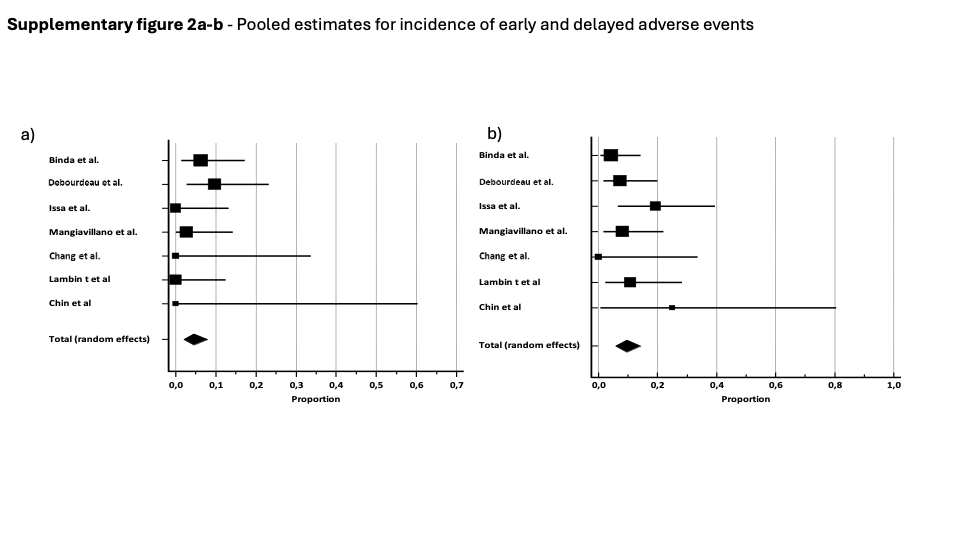

Supplement: Supplementary file 1 [file cancers-17-01983-s001.zip › Supplementary Figure S2a-b.tiff]

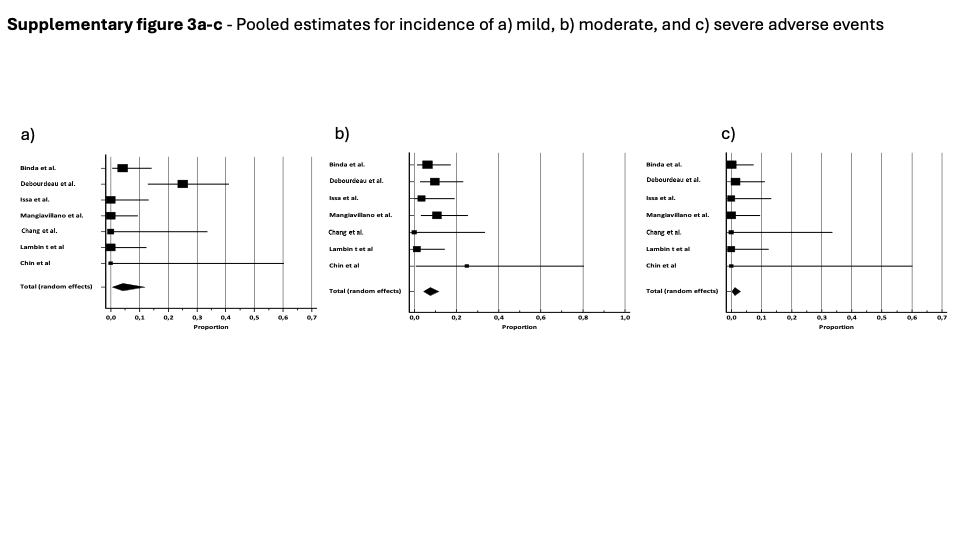

Supplement: Supplementary file 1 [file cancers-17-01983-s001.zip › Supplementary Figure S3a-c.tiff]
